# Trends in influenza- and pneumonia-related mortality in lung cancer patients from 1999 to 2022: a retrospective CDC WONDER analysis

**DOI:** 10.1186/s12931-025-03336-0

**Published:** 2025-09-01

**Authors:** Kate Woods, Mustafa Beidas, Vikram Murugan, Taylor Billion, Abubakar Tauseef, Mohsin Mirza

**Affiliations:** 1 Creighton University School of Medicine, Omaha, NE USA; 2 Department of Internal Medicine, Creighton University, Omaha, NE USA

**Keywords:** Lung cancer, Pneumonia, Influenza, Cancer disparity, Health equity, CDC WONDER

## Abstract

**Background:**

Lung cancer is the most frequent cause of cancer-related deaths in the United States and worldwide. Infectious diseases such as pneumonia and influenza are major risk factors for morbidity and mortality in patients diagnosed with lung cancer. Our study expands upon existing literature investigating epidemiological differences in lung cancer mortality, using the Centers for Disease Control and Prevention (CDC) Wide-ranging Online Data for Epidemiology Research (WONDER) database to report on influenza- and pneumonia-related mortality rates in lung cancer patients through multiple decades.

**Methods:**

CDC WONDER was used to identify influenza- and pneumonia-related deaths in lung cancer patients that occurred within the United States from 1999 to 2022. Crude and age-adjusted mortality rates (AAMR) were calculated, as well as annual percent change and weighted average annual percent change with 95% confidence intervals for the AAMRs. The Joinpoint Regression Program was used to determine trends in mortality within the study period.

**Results:**

From 1999 to 2022, male lung cancer patients demonstrated greater mortality rates from pneumonia and influenza compared to females (60.6% vs. 39.4%). When stratified by race and ethnicity, Black patients had the highest AAMR over the study period at 9.1 per 100,000 people in 1999, as well as the most significant reduction in AAMR to 4.9 per 100,000 people in 2022. Additionally, AAMRs were consistently higher in rural areas compared to urban locations. By age group, patients aged 75–84 had the highest overall crude mortality rate at 28.3 per 100,000 people in 1999, with the lowest rate in ages 35–44 at 0.2 per 100,000 people in 2022.

**Conclusion:**

This study expands upon previously reported trends in lung cancer mortality, highlighting epidemiological differences in influenza- and pneumonia-related death. Significant disparities in mortality rates were noted in older-aged, male, Black, and rural lung cancer patients. Targeted public health strategies concerning the unique needs of these diverse populations will be essential in improving mortality rates. Future research should also investigate the underlying causes of these disparities, and examine how certain community-based initiatives, such as campaigns for tobacco cessation, vaccination, and lung cancer screening, can inform guidelines and reduce preventable mortality in high-risk groups.

**Supplementary information:**

The online version contains supplementary material available at 10.1186/s12931-025-03336-0.

## Introduction

Lung cancer remains the most common cause of cancer-related deaths in the United States and worldwide [1]. The Centers for Disease Control and Prevention (CDC) reports that about 209,000 people in the United States are diagnosed with lung cancer each year, and about 132,000 people die from this disease [2]. The primary risk factor for the development of lung cancer is cigarette smoking, which is linked to 80–90% of lung cancer deaths in the United States [3]. Current trends in mortality of U.S. lung cancer patients have shown a steady decline since 1990, partially due to decreases in cigarette smoking [4]. Factors that have contributed to the reduction in cigarette smoking include prevention efforts and regulating the purchases of tobacco products [4]. However, the complications related to smoking and lung cancer remain prevalent, particularly with infectious diseases. Prior studies have shown that in heavy smokers admitted due to community-acquired pneumonia, there was a higher incidence of subsequent lung cancer rates in the year following admission [5].

Regardless of smoking status, infectious diseases such as pneumonia and influenza remain a significant cause of morbidity and mortality in lung cancer patients [6]. These patients experience an increased susceptibility to infection due to immune suppression, either from the disease itself or as a result of therapeutic interventions [7]. Certain chemotherapeutic medications, such as myelosuppressive agents, can result in neutropenia that further inhibits the ability to fight off infections [7]. Treatment for lung cancer also may cause cytotoxic chemotherapy-induced mucosal injury of the oral cavity and intestinal epithelium [8]. Such injury may increase the risk of colonization by infectious organisms [8]. The propensity for respiratory infections in the lung cancer population has a significant impact on outcomes. Previous research has demonstrated that influenza and pneumonia remain the most common infectious diseases causing death among cancer patients [6], and that cancer patients with influenza have a higher incidence of complications [9]. These complications led to a longer length of stay in comparison to those without influenza [9].

Additional disparities in lung cancer outcomes have been identified in relation to social determinants of health. One study by Theik et al. revealed that more than 40% of lung cancer patients are racial and ethnic minorities, and that disparities exist in screening, treatment, clinical outcomes, and survival rates in these populations [10]. Black patients are disproportionately affected, as they experience the highest incidence of lung cancer, increased rates of advanced-stage disease at diagnosis, and worse overall mortality compared to their White counterparts [11, 12]. There are also racial disparities in treatment, with Black patients less likely than White patients to undergo surgery, lymph node resection, or receive immunotherapy [11, 12]. Socioeconomic status has additionally been shown to impact outcomes in lung cancer patients, with low-income and uninsured patients experiencing worse overall prognosis and mortality [13, 14].

Prior studies have examined various factors associated with outcomes in lung cancer patients, including demographic, socioeconomic, and infectious variables [6, 9, 10]. However, our study aims to provide a more comprehensive analysis of mortality differences in lung cancer patients who contract influenza and pneumonia. We expand upon prior reports of the epidemiological differences in lung cancer mortality, investigating the effects of sex, age, race/ethnicity, rural versus urban status, state of residence, and geographic region on outcomes in this population. We utilize the CDC’s Wide-ranging Online Data for Epidemiology Research (WONDER) database to examine the trends in mortality due to influenza and pneumonia in lung cancer patients through multiple decades.

## Methods

The CDC WONDER database provides mortality data across a variety of conditions and categories. CDC WONDER’s Multiple Cause of Death database was queried to identify deaths in which both lung cancer and influenza or pneumonia were listed on the death certificate within the United States, regardless of whether either was the underlying or a contributing cause [15]. Influenza- and pneumonia-related mortality was identified using the International Classification of Diseases, 10th Revision (ICD-10) codes J09-J18 in patients ≥ 35 years with confirmed cases of lung cancer (ICD-10 codes C34.0-C34.2 and C34.8-C34.9). ICD codes J09-J18 were selected based on CDC guidance for identifying influenza and pneumonia-related deaths in national mortality studies. While these codes are broadly accepted for surveillance, misclassification may occur when multiple causes of death are present on death certificates. Individuals under 35 years of age were excluded in this analysis, as lung cancer incidence in individuals younger than 40 years of age is rare and may produce instability in mortality rate estimates [16]. Additionally, we extracted data on lung cancer patients with influenza/pneumonia listed as contributing or underlying causes of death on their death certificates from 1999 to 2022. Data on demographic and regional groups were extracted, including sex, race/ethnicity, age, urban-rural classification, region, and states. For urban-rural classifications, the National Center for Health Statistics (NCHS) Urban-Rural Classification Scheme was used to divide the population into urban (large metropolitan area [population ≥ 1 million], medium/small metropolitan area [population 50,000 to 999,999]) and rural (population < 50,000) counties per the 2013United States census classification [17]. The NCHS 2013 scheme was applied across the study period, and although it has undergone revisions from prior versions, the classifications have overall remained consistent since 1990 [17]. Regions were classified into Northeast, Midwest, South, and West according to the Census Bureau definitions [18]. This retrospective approach allowed for long-term comprehensive analysis of U.S population mortality trends that would not be feasible using institutional or clinical registry data.

Influenza- and pneumonia-related crude and age-adjusted mortality rates (AAMR) were calculated. Crude mortality rates were calculated by dividing the number of influenza- and pneumonia-related deaths by the corresponding United States population. AAMRs were standardized using the 2000United States standard population. Temporal trends in AAMR were analyzed using the Joinpoint Regression Program (version 5.3.0 available from National Cancer Institute, Bethesda, Maryland) was used to determine trends in mortality within the study period [19]. The number or joinpoints was determined using the Monte Carlo permutation method, which selects the optimal number of statistically significant trend segments that best fit the data. 95% confidence intervals (CIs) for the AAMRs were calculated using standard error estimates provided by the Joinpoint Regression Program. Annual percentage change (APC) and average annual percentage change (AAPC) were calculated for each time segment and for the overall study period. The weighted average of the APCs were calculated through the Joinpoint Regression Program to determine AAPCs and corresponding 95% CIs as a summary of the reported mortality trend for the entire study period. APCs and AAPCs were considered increasing or decreasing if the slope describing the change in mortality over the time interval was significantly different from zero using 2-tailed t-test. Statistical significance was set at *p* ≤ 0.05. Statistically significant values are marked with an asterisk (*) in the results section. Due to the aggregate nature of CDC WONDER data, individual adjustment for confounding variables, including comorbid conditions and treatment modalities, was not possible. However, subgroup analyses across demographic variables were conducted to explore variation within the national population.

## Results

Statistically significant values are marked with an asterisk (*)

### Overall:

From 1999 to 2022, there were 212,321 deaths due to influenza and pneumonia among patients diagnosed with lung cancer in the United States (Supplemental Table S1). Overall age-adjusted mortality rates (AAMR) decreased during this period from 7.5 (95% Confidence Interval (CI): 7.3–7.6) in 1999 to 4.1 (95% CI: 4.0-4.1) in 2022, with an average annual percentage change (AAPC) of −2.5* (95% CI: −2.8 to −2.2) (Supplemental Table S1).

APC by time period was:

1999–2006: APC = −1.9* (95% CI: −2.7 to )

2006–2009: APC = −10.7* (95% CI: −12.1 to −7.1)

2009–2019: APC = −2.1* (95% CI −3.8 to −1.2)

2019–2022: APC = 3.4 (95% CI −0.2-8.1)

**Fig. 1:**
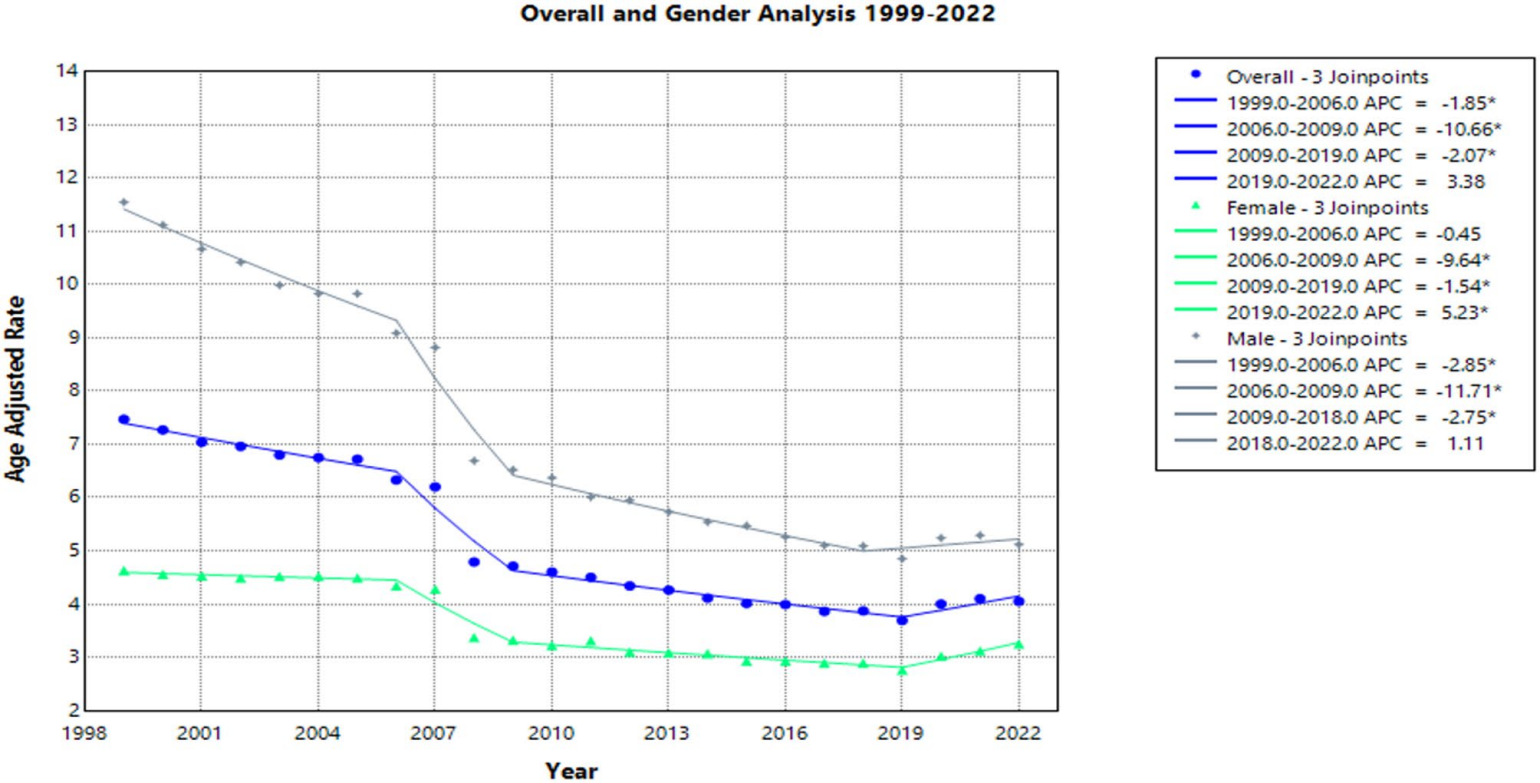
Age-Adjusted Pneumonia and Influenza Mortality Rates in Lung Cancer Patients 1999–2022 Overall and Stratified by Sex

The lowest AAMR was observed in 2019 at 3.69, followed by an increase to 4.1 in 2020. Trends are shown in Fig. 1; full data are provided in Supplemental Table S1.

### Demographic differences:

#### Sex Stratified

From 1999 to 2022, influenza and pneumonia caused 128,709 deaths (60.6%) in male lung cancer patients and 83,612 deaths (39.4%) in female patients in the United States (Supplemental Table S1). During this period, the AAMR in males decreased from 11.6 (95% CI: 11.3–11.8) in 1999 to 5.1 (95% CI: 5.0-5.3) in 2022, with an AAPC of −3.3* (95% CI: −3.7 to −3.1) (Supplemental Table S1).

The APC for males varied across time periods:

1999-2006: APC = -2.8%* (95% CI: -3.8 to -1.6)

2006-2009: APC = -11.7%* (95% CI -13.2 to -4.8)

2009-2018: APC = -2.7* (95% CI -8.2 to -1.8)

2018-2022: APC = 1.1 (95% CI -1.5 to 5.7)

In female lung cancer patients, the AAMR from influenza and pneumonia also declined over the study period, from 4.6 (95% CI 4.5 to 4.8) in 1999 to 3.3 (95% CI 3.2 to 3.4) in 2022. The AAPC was − 1.5* (95% CI −1.7 to −1.2) (Supplemental Table S1).

The APC by time period for females was:

1999-2006: APC = -0.5 (95% CI -1.2 to 0.7)

 2006-2009: APC = -9.6* (95% CI -11.0 to -6.5)

 2009-2019: APC = -1.5* (95% CI -2.4 to -0.7)

 2019-2022: APC = 5.2* (95% CI 2.0 to 10.0)

All the above changes are visualized in Fig. 1, and full APC data for both sexes and all periods is available in Supplemental Table S1.

#### Race stratified

Black or African American individuals had the highest AAMR throughout the study period, and the most significant reduction in AAMR, from 9.1 (95% CI: 8.6–9.6) in 1999 to 4.9 (95% CI: 4.6–5.2) in 2022. The overall AAPC was − 2.6* (95% CI: −2.8 to −2.3) (Supplemental Table S2).

APC by time period for Black/African American patients:

1999-2006: APC = -2.4* (95% CI: -3.3 to -0.8)

 2006-2009: APC = -8.7* (95% CI: -10.3 to -5.4)

 2009-2018: APC = -3.0* (95% CI: -4.0 to -1.6)

 2018-2022: APC = 3.0* (95% CI: 0.6-7.6)

Hispanic individuals consistently had the lowest AAMR among racial and ethnic groups, decreasing from 4.3 (95% CI: 3.8–4.8) in 1999 to 2.5 (95% CI: 2.1–2.6) in 2022. The AAPC was − 2.5* (95% CI:−3.0 to−2.0) (Supplemental Table S2).

APC by time period for Hispanic patients:

1999-2015: APC = -4.6* (95% CI: -5.6 to -3.9)

 2015-2022: APC = 2.5* (95% CI: 0.4-6.3)

Among Asian or Pacific Islander individuals, AAMR decreased from 6.6 (95% CI: 5.7–7.5) in 1999 to 3.5 (95% CI: 3.2–3.8) in 2022. The AAPC was − 3.0* (95% CI: −3.6 to −2.5) (Supplemental Table S2).

APC by time period for Asian or Pacific Islander patients:

1999-2013: APC = -4.4* (95% CI: -6.4 to -3.5)

 2013-2022: APC = -0.8 (95% CI: -2.3-3.4)

The AAMR for American Indian or Alaskan Native populations declined from 7.3 (95% CI: 5.2–9.9) in 1999 to 4.1 (95% CI: 3.1–5.4) in 2022. The AAPC was − 3.2* (95% CI: −4.4 to −2.0) (Supplemental Table S2).

Among White patients, the AAMR fell from 7.5 (95% CI: 7.3–7.7) in 1999 to 4.3 (95% CI: 4.2–4.4) in 2022. The AAPC was − 2.3* (95% CI: −2.6 to −2.0) (Supplemental Table S2).

APC by time period for White patients:

1999-2006: APC = -1.5* (95% CI: -2.4 to -0.2)

 2006-2009: APC = -11.1* (95% CI: -12.7 to -7.1)

 2009-2019: APC = -1.8* (95% CI: -4.2 to -0.9)

 2019-2022: APC = 3.7 (95% CI: -0.2-8.7)

**Fig. 2:**
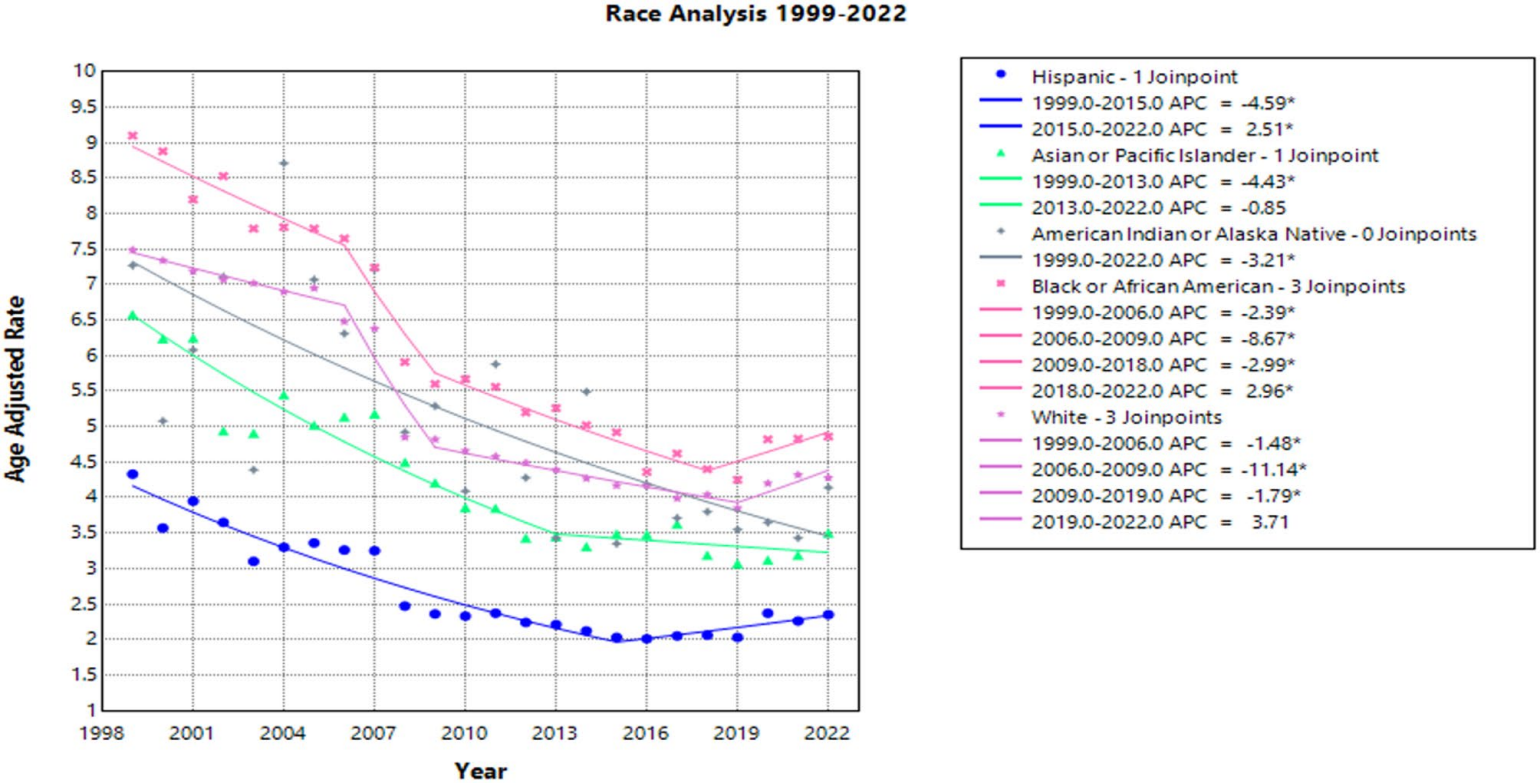
Age-Adjusted Pneumonia and Influenza Mortality Rates in Lung Cancer Patients 1999–2022 Stratified by Race and Ethnicity.

See Fig. 2 and Supplemental Table S2 for detailed trends for all racial and ethnic groups.

### Age group stratified

Age groups were defined as 35–44, 45–54, 55–64, 65–74, 75–84, and 85 + years of age. The population aged 75–84 years had the highest overall crude mortality rate, which decreased from 28.3 (95% CI: 27.3–29.2) in 1999 to 15.2 (95% CI: 14.7–15.8) in 2022. The AAPC was − 2.5* (95% CI: −2.7 to −2.1) (Supplemental Table S3).

APC by time period for patients aged 75–84:

1999-2006: APC = -1.1 (95% CI: -2.1-0.3)

 2006-2009: APC = -11.0* (95% CI: -12.6 to -7.3)

 2009-2018: APC = -2.5* (95% CI: -4.1 to -1.6)

 2018-2022: APC = 2.1 (95% CI: -0.3-6.7)

Individuals aged 35–44 years had the lowest overall crude mortality rate, which declined from 0.3 (95% CI: 0.2–0.3) in 1999 to 0.2 (95% CI: 0.2–0.2) in 2022. The AAPC was − 1.6* (95% CI: −3.3 to −0.8) (Supplemental Table S3).

APC by time period for patients aged 35–44:

1999-2020: APC = -3.5* (95% CI: -6.4 to -2.5)

 2020-2020: APC = 19.9 (95% CI: -3.0-34.2)

**Fig. 3:**
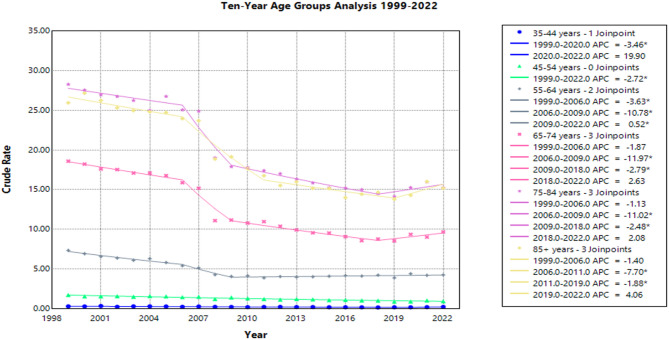
Crude Pneumonia and Influenza Mortality Rates in Lung Cancer Patients 1999–2022 Stratified by Age.

The age cohort 45–54 also had low crude mortality rates of 1.7 (95% CI: 1.6–1.8) in 1999 that further decreased to 0.9 (95% CI: 0.8-1.0) in 2022 (Supplemental Table S3). The AAPC in this group during the study time period was − 2.7* (95% CI: −3.2 to −2.3) (Fig. 3).

The crude mortality rates in the population aged 55–64 declined from 7.3 (95% CI: 7.0-7.7) in 1999 to 4.2 (95% CI: 4.0-4.4) in 2022, with an AAPC of −2.3* (95% CI: −2.5 to −2.0) (Supplemental Table S3).

APC by time period for patients aged 55–64:

1999-2006: APC = -3.6* (95% CI: -4.4 to -2.1)

 2006-2009: APC = -10.8* (95% CI: -12.4 to -7.1)

 2009-2022: APC = 0.5* (95% CI: 0.1-1.1)

For individuals aged 65–74, crude mortality rates fell from 18.6 (95% CI: 18.0-19.2) in 1999 to 9.7 (95% CI: 9.3–10.0) in 2022, with an AAPC of * (95% CI: −3.3 to −2.5) (Fig. 3, Supplemental Table S3).

APC by time period for patients aged 65–74:

1999-2006: APC = -1.9 (95% CI -3.3 to 0.0)

 2006-2009: APC = -12.0* (95% CI -14.0 to -3.4)

 2009-2018: APC = -2.8* (95% CI -7.6 to -1.2)

 2018-2022: APC = 2.6 (95% CI -0.7 to 9.1)

Lastly, for individuals aged 85 and older, the crude mortality rates declined from 26.0 (95% CI: 24.4–27.5) in 1999 to 15.2 (95% CI: 14.2–16.1) in 2022. The AAPC was − 2.3* (95% CI: −2.7 to −1.9) (Fig. 3, Supplemental Table S3).

APC by time period for patients aged 85+:

1999-2006: APC = -1.4 (95% CI: -2.8-0.5)

 2006-2011: APC = -7.7* (95% CI: -11.1 to -2.4)

 2011-2019: APC = -1.9* (95% CI: -7.0 to -0.6)

 2019-2022: APC = 4.1 (95% CI: -0.2-9.5)

These trends by age group are presented in Fig. 3 and detailed in Supplemental Table S3.

### Regional variation:

#### Rural vs. urban

When comparing populated regions, AAMRs were consistently higher in rural areas compared to small, medium, and large metropolitan regions throughout the study period. In rural zones, AAMR decreased from 8.6 (95% CI: 8.3-9.0) in 1999 to 5.0 (95% CI: 4.8–5.2) in 2020, with an AAPC of −2.8* (95% CI −3.1 to −2.4) (Supplemental Table S4).

APC by time period for rural patients:

1999-2006: APC = -1.0 (95% CI: -2.1-0.8)

 2006-2009: APC = -12.1* (95% CI: -14.2 to -7.3)

 2009-2020: APC = -1.3* (95% CI: -2.0 to -0.1)

In urban zones, AAMR decreased from 7.2 (95% CI: 7.0-7.3) in 1999 to 3.8 (95% CI: 3.7–3.9) in 2020, with an AAPC of −3.3* (95% CI: −3.5 to −3.0) (Supplemental Table S4).

APC by time period for urban patients:

1999-2006: APC = -2.0* (95% CI: -2.9 to -0.4)

 2006-2009: APC = -10.6* (95% CI: -12.3 to -6.8)

 2009-2020: APC = -2.0* (95% CI: -2.6 to -1.2)

**Fig. 4:**
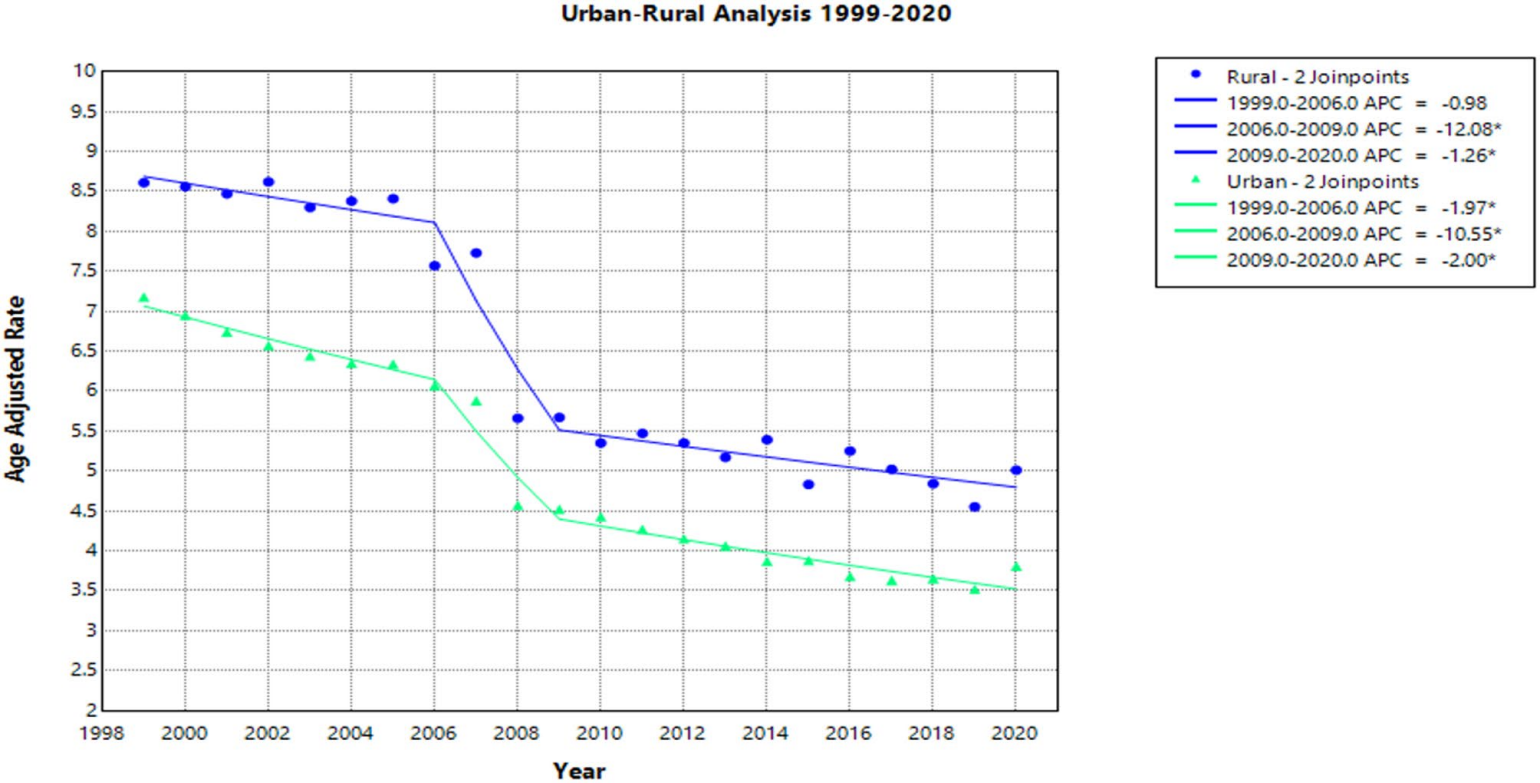
Age-Adjusted Pneumonia and Influenza Mortality Rates in Lung Cancer Patients 1999–2020 Stratified by Rural versus Urban Living.

Refer to Fig. 4 and Supplemental Table S4 for additional details.

#### State-level difference

**Fig. 5:**
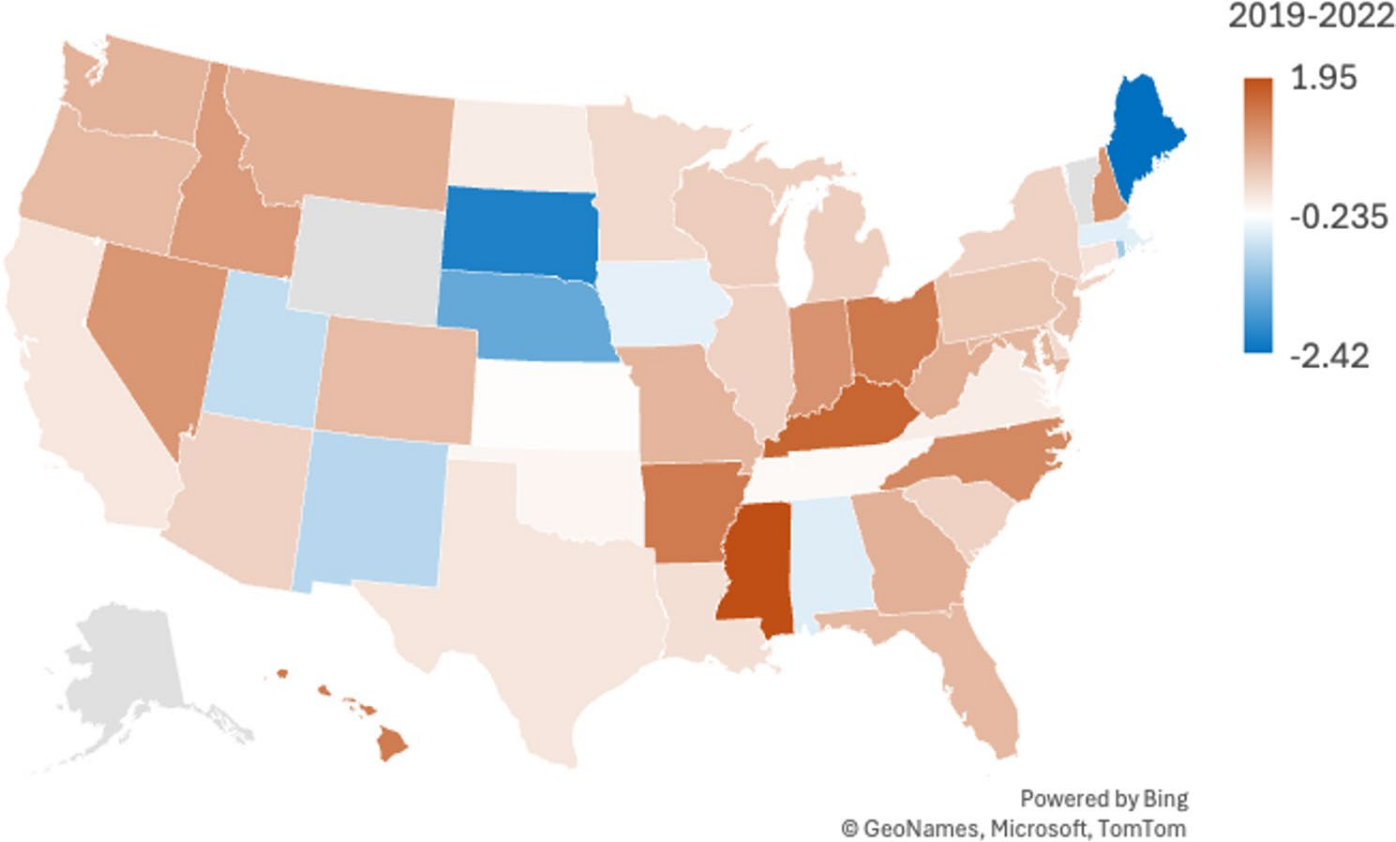
Age-Adjusted Pneumonia and Influenza Mortality Rates in Lung Cancer Patients 2019–2020 Stratified by State of Residence

The state with the largest change in AAMR was Rhode Island, with an AAMR of −10.39 from 1999 to 2019 and − 11.41 from 1999 to 2022 (Supplemental Table S5). Over the entire time period of 1999–2022, no states had a positive change in AAMR, suggesting that mortality from pneumonia and influenza in lung cancer patients is improving nationally with time. The state with the smallest change in AAMR was Indiana with − 1.21 from the years 1999–2022 (Supplemental Table S5). During the years of the COVID-19 pandemic (2019–2022), Maine had the largest change in AAMR at −2.42, North Dakota had the smallest change in AAMR at a value of 0, and Mississippi had the largest positive change in AAMR at 1.95 (Fig. 5) (Supplemental Table S5). Of note, the CDC WONDER database did not have enough reliable data to provide AAMR for Alaska or Wyoming.

#### Census region-based differences

All census regions had a decrease in AAMR over time, particularly in the West, which saw a decrease in AAMR from 7.5 (95% CI: 7.1–7.8) in 1999 to 3.3 (95% CI: 3.2–3.4) in 2022, with an AAPC of −3.3* (95% CI: −3.7 to −3.0) (Supplemental Table S6).

APC by time period for Western region patients:

1999-2006: APC = -2.5* (95% CI: -3.5 to -1.1)

 2006-2009: APC = -11.6* (95% CI: -13.3 to -7.6)

 2009-2019: APC = -3.1* (95% CI: -4.8 to -2.1)

 2019-2022: APC = 2.7 (95% CI: -0.9-7.7)

The Northeast APCs by time period are as follows:

1999-2006: APC = -2.8* (95% CI: -3.1 to -2.4)

 2006-2009: APC = -9.6* (95% CI: -11.7 to -4.9)

 2009-2022: APC = -1.6* (95% CI: -2.3 to -0.1)

In the South, APCs by time period were:

1999-2006: APC = -2.0* (95% CI: -2.9 to -0.7)

 2006-2009: APC = -10.7* (95% CI: -12.2 to -6.9)

 2009-2019: APC = -1.8* (95% CI: -3.9 to -0.8)

 2019-2022: APC = 2.4 (95% CI: 0.0-7.1)

Midwest APCs by time period:

1999-2006: APC = -0.8 (95% CI: -2.1-1.1)

 2006-2009: APC = -11.4* (95% CI: -13.2 to -3.2)

 2009-2019: APC = -1.7* (95% CI: -7.9 to -0.8)

 2019-2022: APC = 4.4 (95% CI: -0.4-10.7)

**Fig. 6:**
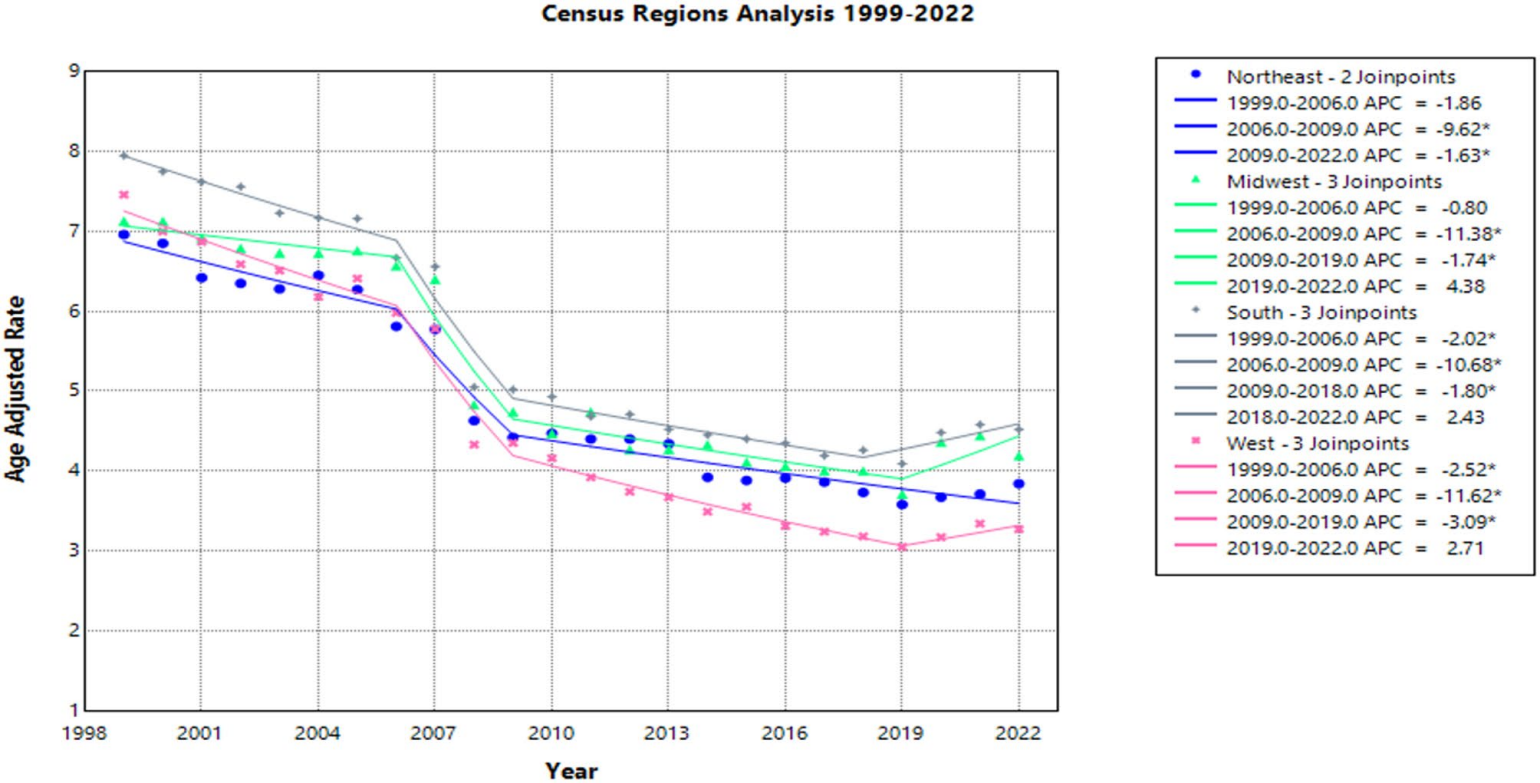
Age-Adjusted Pneumonia and Influenza Mortality Rates in Lung Cancer Patients 1999–2022 Stratified by United States Census Region.

Census region-based differences are illustrated in Fig. 6 and further detailed in Supplemental Table S6.

## Discussion

Lung cancer is the leading cause of cancer-related deaths both in the United States and worldwide, even despite improvements in risk assessment, diagnosis, and treatment [1]. While smoking rates have declined globally, tobacco use remains the primary risk factor for lung cancer development and a significant health burden in low- and middle-income countries [20]. Additionally, lung cancer patients are at an increased risk of developing infectious complications, such as influenza and pneumonia, due to immunosuppressive effects of treatments like chemotherapy [7]. These infections contribute to non-cancer mortality in this patient population, with lung cancer patients experiencing significantly higher rates of influenza- and pneumonia-related death than the general population [21]. This study expands upon existing literature by specifically examining temporal trends in influenza and pneumonia mortality among individuals with lung cancer in the United States across multiple decades.

Overall, our results show declining age-adjusted mortality rates (AAMR) from 1999 to 2022, with the lowest AAMR present in 2019. Patients aged 75–84 had the highest overall crude mortality rate, which decreased from 28.3 per 100,000 people in 1999 to 15.2 per 100,000 people in 2022. The age group with the lowest overall crude mortality rate was ages 35–44. This pattern is consistent with the general epidemiology of lung cancer, as older adults—who often have more comorbidities and less physiological resilience—face greater risk of complications and death from infections like influenza and pneumonia [22, 23]. While younger patients may have more favorable outcomes after cancer diagnosis, our findings reflect broader trends in mortality at the population level rather than individual survival probabilities.

When stratified by sex, we observed increased mortality due to influenza and pneumonia among individuals with lung cancer in males compared to females (60.6% vs. 39.4%). This trend is consistent with prior studies demonstrating a higher incidence of lung cancer in males versus females, often associated with more advanced-stage disease at presentation [24, 25]. While these sex-related differences are likely influenced by a complex interplay of factors, some proposed explanations include variations in sex hormone contributions, immune response, and genetic factors [25]. Estradiol, progesterone, and testosterone have been implicated in the regulation of lung cancer biology, however their exact effects continue to be debated in literature [25]. Immune function also appears to differ by sex, with some evidence suggesting females may mount a more robust immune response [26], potentially enhancing their ability to combat or prevent malignancy. Genetic distinctions have also been identified; for example, lung cancer in females is more frequently associated with mutations in the p53 and cytochrome P450 genes, impacting their ability to repair DNA damage successfully [25]. Additionally, sex-based differences in the prevalence of specific genetic mutations have been documented. KRAS mutations are more common in male smokers with lung cancer, whereas EGFR mutations are more frequent in Asian female never-smokers with lung adenocarcinoma [27]. Females also metabolize nicotine more efficiently than males, largely due to higher estrogen levels that enhance the activity of the CYP2A6 enzyme—an effect that may impact lung cancer development in smokers [25, 28]. Lastly, sociocultural and behavioral factors may further influence outcomes. A systematic review by Rana et al. found that females with lung cancer were more likely to be diagnosed at an earlier stage and were more inclined to utilize inpatient cancer care and surgical treatments compared to males [29]. More broadly, females tend to be more proactive in seeking out cancer-related information than males [30]. These behavioral patterns are often attributed to the “masculinity effect,” wherein men tend to minimize or ignore their healthcare needs and symptoms [31]. Such patterns may contribute to earlier detection and better management of complications such as influenza and pneumonia. However, these factors may influence both the incidence of lung cancer and outcomes after diagnosis. Our observed mortality trends reflect population-level patterns rather than directly measuring individual survival. Thus, further research is necessary to clarify the complex relationships between biological and sociocultural factors on sex-based differences in lung cancer to guide sex-specific strategies for prevention and care.

Mortality rates also varied between racial and ethnic groups, with Black individuals experiencing the highest AAMR over the study period, as well as the most significant reduction in AAMR. In contrast, Hispanic patients consistently had the lowest AAMR compared to the other racial/ethnic cohorts. These trends are reflective of prior literature, which has shown significantly higher rates of all-cause mortality in Black patients with early-stage lung cancer compared to their White counterparts [32]. Racial differences may not be explainable by race alone, however, as one single-center study reported through their multivariable analysis, which controlled for social determinants of health, that Black race itself was not associated with worse overall outcomes [33]. Rather, insurance type and marital status demonstrated worse mortality in the study’s cohort [33].In contrast, Hispanic and Asian patients had improved outcomes compared to White patients due to lower mortality from both lung cancer and all other causes [32]. Interestingly, one study demonstrated that improved mortality in Hispanic patients exists despite a considerably worse risk factor profile [34]. The influence of racial and ethnic disparities on mortality is complex, and factors beyond race, such as socioeconomic determinants, insurance status, and comorbidities likely play a critical role in outcomes.

Despite the increased mortality rates observed in Black patients with lung cancer, the significant reduction in this group’s AAMR over our study period is an encouraging finding. One possible explanation for this reduction is the development of various community-based and culturally sensitive public health interventions aimed at reducing tobacco use in the Black community. Several such initiatives have been implemented at community and state levels, [35–37] and while more data is required to better determine long-term outcomes of these programs, they offer promising alternatives to existing traditional and centralized interventions. Given the predictive risk of tobacco use in the development of lung cancer [3], targeted prevention efforts like these may help mitigate disease incidence and associated mortality. This is especially important for vulnerable populations like racial and ethnic minorities who still experience disparities in lung cancer screening and treatment [38].

AAMRs were also consistently higher in rural areas than in small, medium, and large metropolitan regions. This aligns with prior literature, which demonstrates worse mortality in individuals living in rural versus urban areas. A report from the Economic Research Service with the U.S. Department of Agriculture noted that age-adjusted natural-cause mortality rates were 6% higher in rural areas compared to urban areas in 1999, and this gap grew to 20% in 2019 [39]. Several factors may contribute to these population-level differences, including limited healthcare resources, physician shortages, delayed seeking of care, and a primarily older patient population in rural areas [40]. Additionally, rural communities are disadvantaged by reduced public spending for social services targeting social determinants of health, likely contributing to broader disparities in health outcomes [41]. In this context, the higher AAMRs for rural areas in our study likely reflect increased disease burden and structural barriers to prevention and early intervention in these communities. Addressing these disparities will require endeavors to improve access and quality of healthcare administered to rural areas. One potential strategy is targeted vaccination campaigns, as vaccination coverage for rural areas is consistently lower than urban regions for preventable diseases such as HPV, meningitis, and COVID-19 [42, 43]. Strengthening vaccination outreach in these communities could be influential in reducing preventable mortality and promoting public health outcomes.

The influence of public health infrastructure and policy is reflected in our analysis of state-level trends in AAMRs. Rhode Island experienced the greatest reduction in AAMR over the study period, possibly reflecting the state’s efforts to address social determinants of health and invest in public health infrastructure. The Commonwealth Fund’s annual *Scorecard on State Health System Performance*compares healthcare policies across states, evaluating 40-50 indicators related to healthcare access, quality of care, service use and costs of care, health outcomes, and income-based health care disparities [44]. In the 2019 report, Rhode Island was recognized as the most improved state between 2013 and 2017, having reduced its adult uninsured rate from 17–6% [44]. The state’s progress has continued, ranking among the top five in the 2023 State Scorecard [45]. In contrast, Mississippi has consistently ranked among the lowest-performing states, with the 2022 Scorecard also highlighting its inadequate response to the COVID-19 pandemic [46]. This aligns with our findings that Mississippi had the largest increase in AAMR from 2019 to 2022. A key contributing factor may be the state’s refusal to expand Medicaid under the Affordable Care Act, leaving a significant portion of the population without health coverage and exacerbating health vulnerabilities during the pandemic [45]. Taken together, the correspondence between state-level policy reports and mortality trends emphasize the potential impact of public health and legislative action on population health outcomes.

One notable trend among our analysis is the steep decline in age-adjusted mortality rates between 2006 and 2009, which is consistent across all stratified data. A possible contributing factor to this decline is the development of new lung cancer screening protocols during this period. In 2006, the National Lung Cancer Screening Trial (NLST) demonstrated the significant effectiveness of low-dose computed tomography (LDCT) in detecting early-stage lung cancers and reducing lung cancer-specific mortality, compared to chest radiography [47]. Findings from the NLST may have raised awareness and initiated more frequent implementation of LDCT as a screening tool, and our observed trends in population-level mortality may indirectly capture cumulative impact of earlier detection during this period.

Another important factor to consider regarding the observed trends in our study is the potential impact of the COVID-19 pandemic on outcomes. Since our study focused on influenza- and pneumonia-related deaths, two conditions for which vaccination is available, it is important to examine how the pandemic may have influenced public opinion on vaccines. In fact, multiple studies demonstrated increased vaccine confidence and willingness to receive the influenza vaccine globally during and after the pandemic [48, 49]. However, such changes were not observed in the United States. Influenza vaccine uptake remained relatively stable in the U.S. during the pandemic until the availability of the COVID-19 vaccines became more widespread in the 2021–2022 flu season [50]. After this time, flu vaccination decreased among children but remained relatively stable in older adults [50, 51]. In contrast, pneumococcal vaccines in older individuals aged greater than 65 decreased by 3.3% during the pandemic compared to pre-pandemic CDC data [52]. While this could have influenced pandemic mortality rates in lung cancer patients, in conjunction with the relatively stable flu vaccine rates observed during the pandemic, our observed mortality rates during this time period are likely related to factors other than vaccine status. Rather, it is more likely that the burden of the pandemic itself led to the increased mortality we observed from 2019 to 2022 overall and within various cohorts. The COVID-19 pandemic limited access to health services and exacerbated pre-existing barriers to receiving care [53]. Difficulties in attaining treatment and diagnostic interventions during the pandemic may have contributed to worse outcomes in lung cancer patients. Many lung cancer patients experienced changes in their treatment plans during the pandemic, and the number of lung cancer patients receiving cancer care diminished as well [54]. Thus, the consequences of the COVID-19 negatively impacted the disease course for this group and possibly led to their increased likelihood of death from infectious diseases like pneumonia or influenza.

Our results clearly demonstrate a need to address disparities in outcomes for certain groups of patients with lung cancer. Targeted public health initiatives aimed at these at-risk populations, such as male, Black, elderly, and rural individuals, will be vital in reducing mortality from pneumonia- and influenza-related complications. From a clinical perspective, this underscores the importance of implementing routine vaccination protocols for influenza and pneumococcal disease as part of standard oncologic care, especially for high-risk patients. Multidisciplinary care teams, including social workers, should also be engaged early in the treatment course to help address socioeconomic and logistical barriers to care—such as transportation, cost, and care coordination—that may limit access to preventative services. In fact, multiple studies have reported the benefits of social work services on health and economic outcomes, including better disease control, quality of life, self-determination, and cost savings for both patients and healthcare systems [55, 56]. Healthcare providers should also be trained to recognize and address barriers to care, such as medical mistrust, access issues, and low health literacy, which are more prevalent in underserved communities [57–60]. Policy changes are additionally necessary to ensure equitable access to preventive and screening services, by incentivizing outreach programs in rural and underserved areas. Policies supporting community-based vaccination and tobacco cessation campaigns could further reduce the burden of lung cancer and infections in these at-risk populations.

Our study is not without limitations, including those inherent in retrospective database analyses. For example, there may be missing data from the CDC WONDER database, as it primarily contains mortality data and may not have detailed clinical information such as subtype or severity of lung cancer, type of treatment, or other factors that may additionally impact outcomes. In fact, we were unable to collect data on Alaska or Wyoming for state-level differences in mortality as the data was unavailable in the CDC WONDER database. The database also does not contain data on treatment modalities, comorbidities, or vaccination status, all of which could influence mortality rates in lung cancer patients. Another limitation to consider is the potential for misclassification of cause of death in the CDC WONDER data. Misclassification in coding is a possibility in many database studies and could impact the accuracy of our reported mortality rates. Additionally, as a retrospective analysis, the generalizability of our results are limited.

## Conclusion

This study adds to the existing literature on trends in mortality for lung cancer patients. Our specific emphasis on death from influenza and pneumonia is relevant given how frequently these infections cause death in this population [4]. We report multiple disparities in mortality rates, particularly in older-aged, male, Black, and rural lung cancer patients, emphasizing the importance of targeted public health interventions that address the specific needs of these diverse populations. Future research should investigate the underlying causes of these disparities, such as social determinants of health, differential access to care, and biological factors influencing susceptibility to infection. Additionally, more studies evaluating the effectiveness of tailored community-based campaigns for tobacco cessation, vaccination, and lung cancer screening will be critical to inform evidence-based guidelines that can reduce preventable mortality in high-risk groups.

## Supplementary information

Supplementary Material 1.

## Data Availability

No datasets were generated or analysed during the current study.
