# Supplementary material for: Trends in influenza- and pneumonia-related mortality in lung cancer patients from 1999 to 2022: a retrospective CDC WONDER analysis
Source: Respir Res. 2025 Sep 1;26:267. doi: 10.1186/s12931-025-03336-0 (PMC12403505; doi:10.1186/s12931-025-03336-0)
Supplement: Supplementary file 1 — Supplementary Material 1. [file 12931_2025_3336_MOESM1_ESM.docx]

**Supplemental Tables**

| **Supplemental Table S1. Annual Percent Change (APC) and Average APC (AAPC) in Pneumonia and Influenza Mortality Rates in Lung Cancer Patients from 1999-2022 – Overall and Stratified by Sex** | | | | | | |
| --- | --- | --- | --- | --- | --- | --- |
| **APC** | | | | | | |
| **Cohort** | **Lower Endpoint (Year)** | **Upper Endpoint (Year)** | **APC** | **Lower CI** | **Upper CI** | **P-Value** |
| Overall | 1999 | 2006 | -1.85 | -2.70 | -0.53 | **0.02** |
| Overall | 2006 | 2009 | -10.66 | -12.11 | -7.10 | **0.01** |
| Overall | 2009 | 2019 | -2.07 | -3.84 | -1.20 | **0.002** |
| Overall | 2019 | 2022 | 3.38 | -0.18 | 8.14 | 0.07 |
| Female | 1999 | 2006 | -0.45 | -1.20 | 0.67 | 0.3 |
| Female | 2006 | 2009 | -9.64 | -11.01 | -6.47 | **0.001** |
| Female | 2009 | 2019 | -1.54 | -2.41 | -0.69 | **0.004** |
| Female | 2019 | 2022 | 5.23 | 1.97 | 9.97 | **0.002** |
| Male | 1999 | 2006 | -2.85 | -3.84 | -1.56 | **0.01** |
| Male | 2006 | 2009 | -11.71 | -13.19 | -4.79 | **0.007** |
| Male | 2009 | 2018 | -2.75 | -8.24 | -1.79 | **0.002** |
| Male | 2018 | 2022 | 1.11 | -1.45 | 5.73 | 0.4 |
| **AAPC** | | | | | | |
| **Cohort** | **Lower Endpoint (Year)** | **Upper Endpoint (Year)** | **AAPC** | **Lower CI** | **Upper CI** | **P-Value** |
| Overall | 1999 | 2022 | -2.48 | -2.80 | -2.20 | **< 0.000001** |
| Female | 1999 | 2022 | -1.46 | -1.74 | -1.20 | **< 0.000001** |
| Male | 1999 | 2022 | -3.35 | -3.66 | -3.06 | **< 0.000001** |

Statistically significant P-values (<0.05) are in bold.

| **Supplemental Table S2. Annual Percent Change (APC) and Average APC (AAPC) in Pneumonia and Influenza Mortality Rates in Lung Cancer Patients from 1999-2022 – Stratified by Race and Ethnicity** | | | | | | |
| --- | --- | --- | --- | --- | --- | --- |
| **APC** | | | | | | |
| **Cohort** | **Lower Endpoint (Year)** | **Upper Endpoint (Year)** | **APC** | **Lower CI** | **Upper CI** | **P-Value** |
| Hispanic | 1999 | 2015 | -4.50 | -5.56 | -3.87 | **<0.000001** |
| Hispanic | 2015 | 2022 | 2.51 | 0.45 | 6.28 | **0.02** |
| API | 1999 | 2013 | -4.43 | -6.39 | -3.55 | **0.0008** |
| API | 2013 | 2022 | -0.85 | -2.26 | 3.39 | 0.5 |
| AIAN | 1999 | 2022 | -3.21 | -4.36 | -2.01 | **<0.000001** |
| Black | 1999 | 2006 | -2.39 | -3.28 | -0.84 | **0.01** |
| Black | 2006 | 2009 | -8.67 | -10.29 | -5.38 | **0.001** |
| Black | 2009 | 2018 | -2.99 | -3.99 | -1.58 | **0.004** |
| Black | 2018 | 2022 | 2.96 | 0.60 | 7.61 | **0.01** |
| White | 1999 | 2006 | -1.48 | -2.37 | -0.19 | **0.04** |
| White | 2006 | 2009 | -11.14 | -12.65 | -7.12 | **0.02** |
| White | 2009 | 2019 | -1.79 | -4.16 | -0.85 | **0.004** |
| White | 2019 | 2022 | 3.71 | -0.21 | 8.72 | 0.07 |
| **AAPC** | | | | | | |
| **Cohort** | **Lower Endpoint (Year)** | **Upper Endpoint (Year)** | **AAPC** | **Lower CI** | **Upper CI** | **P-Value** |
| Hispanic | 1999 | 2022 | -2.48 | -2.97 | -2.00 | **< 0.000001** |
| API | 1999 | 2022 | -3.05 | -3.56 | -2.51 | **< 0.000001** |
| AIAN | 1999 | 2022 | -3.21 | -4.36 | -2.01 | **< 0.000001** |
| Black | 1999 | 2022 | -2.56 | -2.84 | -2.26 | **< 0.000001** |
| White | 1999 | 2022 | -2.28 | -2.63 | -1.98 | **< 0.000001** |

Statistically significant P-values (<0.05) are in bold.

AIAN American Indian/Alaska Native; API Asian or Pacific Islander.

| **Supplemental Table S3. Annual Percent Change (APC) and Average APC (AAPC) in Pneumonia and Influenza Mortality Rates in Lung Cancer Patients from 1999-2022 – Stratified by Age** | | | | | | |
| --- | --- | --- | --- | --- | --- | --- |
| **APC** | | | | | | |
| **Cohort** | **Lower Endpoint (Year)** | **Upper Endpoint (Year)** | **APC** | **Lower CI** | **Upper CI** | **P-Value** |
| 35-44 | 1999 | 2020 | -3.46 | -6.41 | -2.46 | **0.03** |
| 35-44 | 2020 | 2022 | 19.90 | -3.00 | 34.22 | 0.2 |
| 45-54 | 1999 | 2022 | -2.72 | -3.18 | -2.34 | **< 0.000001** |
| 55-64 | 1999 | 2006 | -3.63 | -4.43 | -2.07 | **0.009** |
| 55-64 | 2006 | 2009 | -10.78 | -12.40 | -7.07 | **0.006** |
| 55-64 | 2009 | 2022 | 0.52 | 0.073 | 1.10 | **0.03** |
| 65-74 | 1999 | 2006 | -1.87 | -3.30 | 0.02 | 0.05 |
| 65-74 | 2006 | 2009 | -11.97 | -13.94 | -3.40 | **0.03** |
| 65-74 | 2009 | 2018 | -2.79 | -7.58 | -1.19 | **0.006** |
| 65-74 | 2018 | 2022 | 2.63 | -0.74 | 9.11 | **0.1** |
| 75-84 | 1999 | 2006 | -1.13 | -2.05 | 0.25 | 0.08 |
| 75-84 | 2006 | 2009 | -11.02 | -12.59 | -7.26 | **0.01** |
| 75-84 | 2009 | 2018 | -2.48 | -4.06 | -1.56 | **0.0008** |
| 75-84 | 2018 | 2022 | 2.08 | -0.31 | 6.71 | 0.08 |
| 85+ | 1999 | 2006 | -1.40 | -2.84 | 0.45 | 0.09 |
| 85+ | 2006 | 2011 | -7.70 | -11.13 | -2.38 | **0.03** |
| 85+ | 2011 | 2019 | -1.88 | -6.97 | -0.56 | **0.01** |
| 85+ | 2019 | 2022 | 4.06 | -0.24 | 9.54 | 0.07 |
| **AAPC** | | | | | | |
| **Cohort** | **Lower Endpoint (Year)** | **Upper Endpoint (Year)** | **AAPC** | **Lower CI** | **Upper CI** | **P-Value** |
| 35-44 | 1999 | 2022 | -1.63 | -3.34 | -0.84 | **< 0.000001** |
| 45-54 | 1999 | 2022 | -2.72 | -3.18 | -2.34 | **< 0.000001** |
| 55-64 | 1999 | 2022 | -2.29 | -2.51 | -2.02 | **< 0.000001** |
| 65-74 | 1999 | 2022 | -2.85 | -3.27 | -2.49 | **< 0.000001** |
| 75-84 | 1999 | 2022 | -2.46 | -2.74 | -2.15 | **< 0.000001** |
| 85+ | 1999 | 2022 | -2.28 | -2.68 | -1.95 | **< 0.000001** |

Statistically significant P-values (<0.05) are in bold.

| **Supplemental Table S4. Annual Percent Change (APC) and Average APC (AAPC) in Pneumonia and Influenza Mortality Rates in Lung Cancer Patients from 1999-2022 – Stratified by Rural versus Urban Living** | | | | | | |
| --- | --- | --- | --- | --- | --- | --- |
| **APC** | | | | | | |
| **Cohort** | **Lower Endpoint (Year)** | **Upper Endpoint (Year)** | **APC** | **Lower CI** | **Upper CI** | **P-Value** |
| Rural | 1999 | 2006 | -0.98 | -2.08 | 0.78 | 0.2 |
| Rural | 2006 | 2009 | -12.08 | -14.16 | -7.32 | **< 0.000001** |
| Rural | 2009 | 2020 | -1.26 | -1.99 | -0.13 | **0.03** |
| Urban | 1999 | 2006 | -1.97 | -2.88 | -0.39 | **0.03** |
| Urban | 2006 | 2009 | -10.55 | -12.26 | -6.76 | **< 0.000001** |
| Urban | 2009 | 2020 | -2.00 | -2.55 | -1.19 | **0.0012** |
| **AAPC** | | | | | | |
| **Cohort** | **Lower Endpoint (Year)** | **Upper Endpoint (Year)** | **AAPC** | **Lower CI** | **Upper CI** | **P-Value** |
| Rural | 1999 | 2020 | -2.79 | -3.11 | -2.40 | **< 0.000001** |
| Urban | 1999 | 2020 | -3.26 | -3.52 | -2.96 | **< 0.000001** |

Statistically significant P-values (<0.05) are in bold.

| **Supplemental Table S5. Age-Adjusted Mortality Rate (AAMR) from Pneumonia and Influenza in Lung Cancer Patients from 1999-2022 – Stratified by State of Residence** | | | | |
| --- | --- | --- | --- | --- |
| **AAMR** | | | | |
| **State** | **1999-2019** | **2019-2022** | **1999-2020** | **1999-2022** |
| Alabama | -3.44 | -0.51 | -3.11 | -3.95 |
| Arizona | -1.93 | 0.32 | -1.35 | -1.61 |
| Arkansas | -2.68 | 1.40 | -2.05 | -1.28 |
| California | -6.07 | 0.06 | -6.19 | -6.01 |
| Colorado | -2.64 | 0.60 | -1.81 | -2.04 |
| Connecticut | -2.77 | 0.12 | -2.69 | -2.65 |
| Delaware | -5.48 | 0.23 | -6.08 | -5.25 |
| District of Columbia | -4.22 | – | -3.28 | – |
| Florida | -2.29 | 0.63 | -1.84 | -1.66 |
| Georgia | -3.73 | 0.73 | -2.82 | -3.00 |
| Hawaii | -5.76 | 1.39 | -5.68 | -4.37 |
| Idaho | -4.16 | 0.99 | -3.49 | -3.17 |
| Illinois | -4.56 | 0.32 | -3.60 | -4.24 |
| Indiana | -2.31 | 1.10 | -1.25 | -1.21 |
| Iowa | -2.81 | -0.46 | -3.23 | -3.27 |
| Kansas | -2.49 | -0.21 | -1.78 | -2.70 |
| Kentucky | -4.76 | 1.67 | -2.53 | -3.09 |
| Louisiana | -3.57 | 0.16 | -2.83 | -3.41 |
| Maine | -1.09 | -2.42 | -1.38 | -3.51 |
| Maryland | -7.72 | 0.66 | -7.57 | -7.06 |
| Massachusetts | -4.71 | -0.48 | -4.98 | -5.19 |
| Michigan | -2.87 | 0.36 | -2.59 | -2.51 |
| Minnesota | -2.08 | 0.23 | -1.27 | -1.85 |
| Mississippi | -4.33 | 1.95 | -2.88 | -2.38 |
| Missouri | -4.60 | 0.68 | -3.62 | -3.92 |
| Montana | -3.79 | 0.75 | -2.70 | -3.04 |
| Nebraska | -2.15 | -1.55 | -2.84 | -3.70 |
| Nevada | -2.89 | 1.06 | -1.96 | -1.83 |
| New Hampshire | -2.89 | 1.07 | -3.09 | -1.82 |
| New Jersey | -3.99 | 0.55 | -3.52 | -3.44 |
| New Mexico | -1.18 | -0.84 | -1.12 | -2.02 |
| New York | -2.49 | 0.32 | -2.19 | -2.17 |
| North Carolina | -4.37 | 1.22 | -4.42 | -3.15 |
| North Dakota | -3.28 | 0 | 0.45 | -3.28 |
| Ohio | -4.29 | 1.43 | -3.63 | -2.86 |
| Oklahoma | -2.00 | -0.12 | -2.84 | -2.12 |
| Oregon | -2.09 | 0.61 | -2.18 | -1.48 |
| Pennsylvania | -3.05 | 0.49 | -3.43 | -2.56 |
| Rhode Island | -10.39 | -1.02 | -10.39 | -11.41 |
| South Carolina | -2.93 | 0.32 | -2.99 | -2.61 |
| South Dakota | -1.83 | -2.16 | -4.11 | -3.99 |
| Tennessee | -5.56 | -0.16 | -4.40 | -5.72 |
| Texas | -3.64 | 0.08 | -3.44 | -3.56 |
| Utah | -1.75 | -0.76 | -2.01 | -2.51 |
| Vermont | – | – | – | -5.41 |
| Virginia | -4.81 | -0.01 | -5.13 | -4.82 |
| Washington | -2.62 | 0.71 | -2.71 | -1.91 |
| West Virginia | -7.42 | 0.77 | -8.03 | -6.65 |
| Wisconsin | -2.56 | 0.38 | -2.03 | -2.18 |

Data that was unavailable in the CDC WONDER database are indicated by a dashed line (–).

| **Supplemental Table S6. Annual Percent Change (APC) and Average APC (AAPC) in Pneumonia and Influenza Mortality Rates in Lung Cancer Patients from 1999-2022 – Stratified by United States Census Region** | | | | | | |
| --- | --- | --- | --- | --- | --- | --- |
| **APC** | | | | | | |
| **Cohort** | **Lower Endpoint (Year)** | **Upper Endpoint (Year)** | **APC** | **Lower CI** | **Upper CI** | **P-Value** |
| Northeast | 1999 | 2006 | -1.86 | -2.97 | 0.69 | 0.09 |
| Northeast | 2006 | 2009 | -9.62 | -11.74 | -4.85 | **0.008** |
| Northeast | 2009 | 2022 | -1.63 | -2.33 | -0.13 | **0.04** |
| Midwest | 1999 | 2006 | -0.80 | -2.14 | 1.05 | 0.3 |
| Midwest | 2006 | 2009 | -11.38 | -13.22 | -3.22 | **0.03** |
| Midwest | 2009 | 2019 | -1.74 | -7.87 | -0.76 | **0.004** |
| Midwest | 2019 | 2022 | 4.38 | -0.36 | 10.68 | 0.07 |
| South | 1999 | 2006 | -2.02 | -2.93 | -0.69 | **0.02** |
| South | 2006 | 2009 | -10.68 | -12.24 | -6.90 | **0.01** |
| South | 2009 | 2018 | -1.80 | -3.94 | -0.77 | **0.004** |
| South | 2018 | 2022 | 2.43 | -0.01 | 7.01 | 0.05 |
| West | 1999 | 2006 | -2.52 | -3.52 | -1.06 | **0.01** |
| West | 2006 | 2009 | -11.62 | -13.26 | -7.61 | **0.009** |
| West | 2009 | 2019 | -3.09 | -4.76 | -2.07 | **0.001** |
| West | 2019 | 2022 | 2.71 | -0.86 | 7.72 | 0.1 |
| **AAPC** | | | | | | |
| **Cohort** | **Lower Endpoint (Year)** | **Upper Endpoint (Year)** | **AAPC** | **Lower CI** | **Upper CI** | **P-Value** |
| Northeast | 1999 | 2022 | -2.78 | -3.08 | -2.35 | **< 0.000001** |
| Midwest | 1999 | 2022 | -2.00 | -2.44 | -1.61 | **< 0.000001** |
| South | 1999 | 2022 | -2.36 | -2.64 | -2.07 | **< 0.000001** |
| West | 1999 | 2022 | -3.35 | -3.65 | -3.05 | **< 0.000001** |

Statistically significant P-values (<0.05) are in bold.
